# Global Preventive and Management Strategies and Their Effectiveness in Patients With Secondary Lymphedema: A Scoping Review

**DOI:** 10.7759/cureus.83627

**Published:** 2025-05-07

**Authors:** Vicent Mwesigye, Daniel Atwine, Esther Faith Munguciada, Alice Tillema, Joanita Berytah Tebulwa, Bosco Bekita Agaba, Joel Bazira, Francis Bajunirwe, Itabangi Herbaert, Frederick Byarugaba, Edgar Mulogo

**Affiliations:** 1 Department of Medical Laboratory Science, Mbarara University of Science and Technology, Mbarara, UGA; 2 Department of Research Services, Soar Research Foundation, Mbarara, UGA; 3 Department of Clinical Research, Soar Research Foundation (SRF) Research and Training Centre, Mbarara, UGA; 4 Department of Information and Library Services, Radboud University, Nijmegen, NLD; 5 Department of Obstetrics and Gynecology, Mbarara University of Science and Technology, Mbarara, UGA; 6 Department of Microbiology and Parasitology, Mbarara University of Science and Technology, Mbarara, UGA; 7 Department of Community Health, Mbarara University of Science and Technology, Mbarara, UGA; 8 Department of Microbiology, Busitema University, Busitema, UGA

**Keywords:** filariasis, kamwenge, lymphedema, neglected tropical diseases, podoconiosis, uganda

## Abstract

Secondary lymphedema arises from lymphatic damage, often due to infections, inflammatory conditions, cancer treatments, or trauma, leading to fluid retention, swelling, and mobility issues that impact the quality of life. This scoping review examined global preventive and management strategies for secondary lymphedema, assessing their effectiveness and feasibility, particularly in low-resource settings. Additionally, it explored causative agents and knowledge gaps among patients and healthcare workers in endemic communities.

This review followed the Joanna Briggs Institute (JBI) methodology and reported findings using the Preferred Reporting Items for Systematic Reviews and Meta-Analyses for Scoping Reviews (PRISMA-ScR) guidelines. The search strategy targeted four databases, including PubMed, Embase, Web of Science, and African Index Medicus. The inclusion criteria included all studies that focused on lower or upper limb secondary lymphedema, and reported on either preventive or management interventions or strategies globally, and were published between 2014 and 2024 in English. Data extraction involved two reviewers, with discrepancies resolved through discussion. A descriptive and narrative synthesis was performed to highlight preventive and management strategies and their effectiveness.

A total of 3192 published articles were retrieved from searches of the selected databases, that is, PubMed (n=1467), Embase conference (n=386), Embase articles (n=550), Web of Science (n=713), and Africa Index Medicus (n=76). After the elimination of duplicate and ineligible articles, 45 articles were included in the final qualitative synthesis. Microsurgical techniques are more effective than conservative treatments. Self-care and hygiene-based strategies were more widely used for filarial lymphedema and podoconiosis in endemic areas, whereas the Mass Drug Administration (MDA) and Morbidity Management and Disability Prevention (MMDP) programs had limited success in halting disease progression. Adjunct therapies, including low-level laser therapy, cryotherapy, and kinesio taping, have shown potential in enhancing limb function and quality of life. The primary causes of secondary lymphedema vary geographically, with lymphatic filariasis dominant in tropical regions, podoconiosis in volcanic highlands, and cancer-related lymphedema in high-income areas. Knowledge gaps and misconceptions among patients, healthcare workers, and communities hinder effective disease management, with stigma and misinformation remaining key barriers despite educational efforts.

While microsurgical interventions offer quick, effective clinical outcomes, hygiene-based interventions and self-care practices are the most scalable strategies for the treatment and management of filarial and podoconiosis-related lymphedema in low-resource settings.

## Introduction and background

Lymphedema is a condition with multiple causes that results in the retention of lymphatic fluid in the interstitium caused by lymphatic malfunction [1]. This condition is broadly classified as secondary or primary on the basis of its aetiology. Unlike secondary lymphedema, which results from disease or injury to the lymphatic system, primary lymphedema is attributed to intrinsic lymphatic abnormalities [2]. Primary lymphedema has been reported to be idiopathic in 70% of patients who have no currently identified genetic associations [2]; however, for the other 30% of patients, there is a strong genetic association with approximately 20 different genes linked to lymphatic anomalies [3]. Lymphedema also manifests in genetic syndromes like tuberous sclerosis, Noonan syndrome, Turner syndrome, and capillary malformation arteriovenous malformation syndrome [4].

Secondary lymphedema, which is the focus of this study, is a result of an acquired defect in the lymphatic system. The most prevalent causes of secondary lymphedema are infection, inflammation, malignancy, malignancy-associated therapeutic interventions, trauma, obesity, and immobility [5,6]. The principal cause of lymphedema in tropical countries is lymphatic filariasis due to the mosquito-transmitted parasitic nematodes, *Wuchereria bancrofti*, *Brugia malayi,* and *B. timori* in Asia [7]. These parasitic nematodes travel to the lymphatic system and impede lymphatic flow [5,7]. Long-term infection can induce lymphatic system impairment, which results in significant swelling of the limbs and subsequently elephantiasis or hydrocele [8]. As of 2018, there were an estimated 51 million people living with lymphatic filariasis, with approximately 10 million in the African region [8].

The second most prevalent cause of secondary lymphedema in the tropics is podoconiosis [7]. It is an inflammatory condition that results in obstruction of the lymphatic system from the accumulation of microsilica particles that make their way into the body and lymphatic system via the feet after prolonged contact with soil with high silica concentrations [7,9]. This phenomenon is particularly prevalent in regions with volcanic soils [7]. Podoconiosis is reported to be endemic in 32 countries, 18 of which are in Africa. The reported prevalence of podoconiosis in Africa varies across countries, for example, 0.10% in Kamwenge, Western Uganda [10], 8.08% in Cameroon [11], 7.45% in Ethiopia, 3.87% in Kenya, 2.51% in Tanzania and 4.52% in the volcanic mountainous region of Kapchorwa in Uganda [12].

Secondary lymphedema can also be attributed to a plethora of other causes that damage or obstruct the lymphatic system, including, cellulitis and lymphatic inflammation, lymphogranuloma venereum, as in tuberculosis, malignant disease, large tumors, metastases, treatment for malignancy, trauma and lymphatic tissue damage, lymph node excision, varicose vein surgery, rheumatoid arthritis, obesity, immobility and pretibial myxedema [6]. In developed countries, secondary lymphedema occurs most frequently following cancer-related treatment interventions, particularly those for breast, head and neck, and gynecological cancers [5].

Because there are no curative treatments for either primary or secondary lymphedema, the current care of lymphedema is essentially palliative and focused on controlling progression and complications [4,5]. However, both management and preventive strategies are dependent on the cause of secondary lymphedema, which perception forms the basis of this scoping review.

There have been tremendous efforts to eliminate lymphatic filariasis, which is the most common cause of lymphedema in the tropics. The most notable is the Global Program to Eliminate Lymphatic Filariasis (GPELP), which was launched by the WHO in 2000 in a bid to eradicate lymphatic filariasis globally by 2020 [13]. The strategy adopted by this program involved interrupting transmission and reducing the morbidity of lymphatic filariasis. Interrupting transmission involved Mass Drug Administration (MDA) in endemic areas, which was then followed by Morbidity Management and Disability Prevention (MMDP) [13,14]. The MDA with recommended oral regimens of the antihelmintic medicines albendazole, either alone or with ivermectin, or diethylcarbamazine citrate and albendazole, or a combination of all three, depending on the setting, was implemented in endemic regions [8,13]. Among the 73 countries identified by WHO as endemic for lymphatic filariasis, 18 countries, including Uganda, have completed MDA and are conducting surveillance to validate elimination [13,15].

There have also been global efforts towards the elimination of podoconiosis, and essential to these efforts is the mapping of endemic areas and the ongoing development of the global atlas of podoconiosis [16]. The key strategy for podoconiosis control is prevention of contact with soil, which can be achieved through interventions such as footwear and lymphoedema morbidity management, which could focus on hygiene [17]. Although control and elimination of podoconiosis should be easily achievable, given the absence of a biological agent and vector, a significant challenge to elimination efforts is the lack of awareness that the condition exists and that its aetiology is different from other causes of lymphedema in the tropics [17]. Community engagement and consequently increased awareness of lymphatic filariasis have been vital to efforts to eliminate the condition as a public health problem in different regions globally, resulting in overall health literacy about vector control, hygiene, and care for individuals with lymphedema and other health issues [18]. However, several studies have reported a general lack of awareness of podoconiosis among health workers, policy makers, academics, and communities in endemic areas [19-22].

This scoping review aimed to explore and document the existing preventive and management strategies globally, as well as their effectiveness in patients with secondary lymphoedema, based on a scoping review of published articles [23].

The main review question was: What are the global preventive and management strategies, and their effectiveness in patients with secondary lymphedema based on a scoping review of published articles? Sub-questions included: What are the causative agents of lower and upper limb secondary lymphedema globally? What is the documented level of knowledge and perceptions of patients regarding secondary lymphedema? What is the feasibility of implementing existing preventive and management strategies for secondary lymphedema within low- and middle-income settings?

## Review

This scoping review was conducted using the Joanna Briggs Institute (JBI) methodology [24], and the reporting followed the guidelines of the Preferred Reporting Items for Systematic reviews and Meta-Analyses (PRISMA) extension for Scoping Reviews (PRISMA-ScR) [24]. The scoping review protocol was registered on Open Science Framework (registration DOI: https://doi.org/10.17605/OSF.IO/8YKST).

Search strategy

The method adopted for identifying literature in this scoping study was iterative and focused on achieving in-depth and broad results. We did not impose strict limitations on the search terms, identification of relevant studies, or study selection at the beginning of the literature search. The search strategy aimed to locate all relevant published documents or articles. A three-step search strategy was utilized in this review.

Step 1

An initial limited search of MEDLINE (PubMed) as appropriate was undertaken to identify relevant articles, including previous systematic or scoping review articles on the topic. The text words contained in the titles, abstracts of relevant articles, and index terms used to describe the articles were used to develop a full search strategy.

Step 2

We examined the references of studies included in the systematic review, or references of systematic reviews on the same or similar topic, to identify additional search terms to include in our search strategy.

Step 3

The final search strategies, including all identified keywords and index terms, were developed and adapted for each included database and/or information source. The reference lists of all included study articles were screened for additional studies.

Through database searches for scientific papers and gray literature, records pertinent to the study's aims were gathered. Specifically, we searched the following databases: PubMed, Embase, Web of Science, and African Index Medicus.

Inclusion criteria

This review considered published documents reporting on preventative or management strategies for secondary lymphedema of either the lower or upper limb. The published articles of interest included, but were not limited to, journal articles, international reports, or guidelines. No limitation in the age of the study participants involved in individual studies was considered in article selection. All study designs were eligible, e.g., case reports, cohort studies, case-control studies, cross-sectional studies, randomized controlled trials (RCTs), and reviews. Articles reporting on knowledge of patients, healthcare workers, and the community about secondary lymphedema were also included. The context of the review was global with a focus on articles published between 2014 and 2024 and in English. This time frame allowed us to extract the most recent evidence that could describe current prevention and management practices regarding secondary lymphedema. We also chose a 10-year period, taking into consideration that the secondary lymphedema prevalent in low-resource settings is classified as a neglected tropical disease.

In addition, qualitative studies that are normally excluded from systematic reviews were also considered to enable documentation of the knowledge, attitudes, and acceptability of different secondary lymphedema preventive and management interventions, hence, enabling us to answer the feasibility question.

Through Boolean operators, articles were searched with keywords and mesh terms, specifically restricted to studies that evaluated at least one preventive or management strategy of secondary lymphedema. We used the following search strategies based on source or database:

PubMed Search Strategy

("Elephantiasis"[Mesh] OR "Elephantiasis, Filarial"[Mesh] OR "Non-Filarial Lymphedema"[Mesh] OR filarial [tiab] OR Filarias*[tiab] OR bancrofti*[tiab] OR Brugian[tiab] OR Malayi[tiab] OR podoconios*[tiab] OR nonfilarial[tiab] OR elephantias*[tiab] OR mossy foot[tiab] OR mossy feet[tiab] OR limb swelling*[tiab] OR secondary lymph*edem*[tiab]) AND ("Secondary Prevention"[Mesh] OR "Primary Prevention"[Mesh] OR "prevention and control" [Subheading] OR prevent*[tiab] OR management [tiab])

Embase

(Exp elephantiasis/ OR filarial.ti,ab,kf. OR Filarias*.ti,ab,kf. OR bancrofti*.ti,ab,kf. OR Brugian.ti,ab,kf. OR Malayi.ti,ab,kf. OR podoconios*.ti,ab,kf. OR nonfilarial.ti,ab,kf. OR elephantias*.ti,ab,kf. OR mossy foot.ti,ab,kf. OR mossy feet.ti,ab,kf. OR limb swelling*.ti,ab,kf. OR secondary lymph*edem*.ti,ab,kf.) AND (primary prevention/ or secondary prevention/ OR "prevention and control"/ OR pc.fs. OR prevent*.ti,ab,kf. OR management.ti,ab,kf.)

Web of Science

TS=(( filarial OR Filarias* OR bancrofti* OR Brugian OR Malayi OR podoconios* OR nonfilarial OR elephantias* OR “mossy foot” OR “mossy feet” OR “limb swelling*” OR “secondary lymph*edem*") AND (prevent* OR management))

African Index Medicus

elephantiasis OR filarial OR Filariasis OR bancroftian OR Brugian OR Malayi OR podoconiosis OR nonfilarial OR elephantiasis OR “mossy foot” OR “mossy feet” OR “limb swelling” OR “secondary lymphedema” OR “secondary lymphaedema”

Study/source of evidence selection

Following the search, all identified citations were collated and uploaded to Mendeley (Elsevier, London, UK), and duplicates were removed. Following a pilot test, titles and abstracts were then screened by two independent reviewers for assessment against the inclusion criteria for the review. The full texts of the selected studies were assessed in detail against the inclusion criteria by two independent reviewers. The reasons for the exclusion of sources of evidence from the full text that did not meet the inclusion criteria were recorded and reported in the scoping review. Any disagreements between the reviewers at each stage of the selection process were resolved through discussion or with an additional reviewer. The results of the search and the study inclusion process are reported and presented in a PRISMA flow diagram [25] as part of the review findings.

Data extraction

Data were extracted from the full-text articles included in the scoping review by two independent reviewers using a data extraction tool developed by the reviewers by adapting the JBI data extraction instrument for scoping reviews [26]. A third reviewer acted as the tiebreaker in case of disagreement between the two reviewers, and their review was performed following satisfactory discussions. All discrepancies were discussed and resolved by consensus between the three reviewers. We pilot tested the data extraction form and modified it accordingly before use. The data extraction tool was continually modified and revised as necessary during the process of extracting data from each included published article until the final one was obtained.

We extracted the following data: studies’ characteristics (author name, publication year, country where data were collected, affected limb) and all data related to the primary and secondary outcomes. The data extracted included specific details about the participants, context, study methods, and key findings relevant to the review question/s.

Data analysis and presentation

The data were entered into a Microsoft Excel (Microsoft® Corp., Redmond, WA, USA) spreadsheet and analyzed. We performed a descriptive presentation of the included studies’ characteristics. We also followed the standard results presentation as per PRISMA 2020 guidelines. The primary analysis focused on documenting the existing preventive and management measures for secondary lymphedema and their effectiveness. A secondary analysis was performed to document the causative agents, knowledge on secondary lymphedema, and feasibility of the preventive and management interventions for secondary lymphedema of the lower and upper limbs. A narrative approach to synthesis was used to combine the evidence from the qualitative studies with the quantitative data. Major themes were extracted to explore the similarities, differences, and relationships between the reviews as suggested by other authors, such as the synthesis without meta-analysis (SWiM) guidance on the conduct of narrative syntheses, and the Preferred Reporting Items for Overviews of Reviews guidelines [24].

Search findings

**Figure 1:**
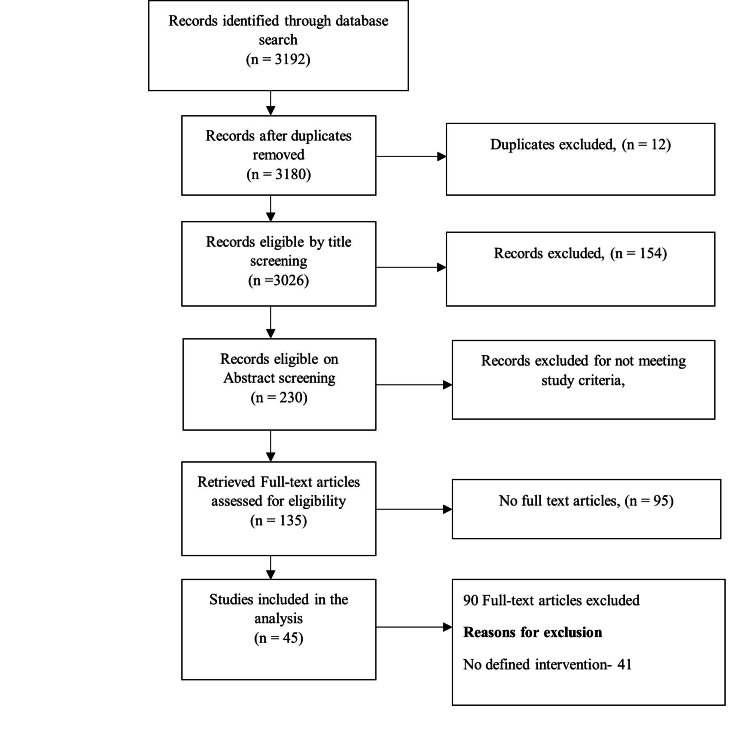
PRISMA flow diagram of article selection PRISMA: Preferred Reporting Items for Systematic Reviews and Meta-Analyses

A total of 3192 published articles were retrieved from all searches made in different databases, including PubMed (n=1467), Embase conference (n=386), Embase articles (n=550), Web of Science (n=713), and Africa Index Medicus (n=76). After the elimination of duplicate articles and those that did not meet the eligibility criteria for title, abstract, or full text screening, and those with irretrievable full texts, 45 articles were included in the final qualitative synthesis or analysis (Figure 1).

Characteristics of the studies

The 45 studies included in this scoping review included 40 individual single-country studies (including one systematic review) and five systematic reviews involving multiple countries, one of which included a meta-analysis. The included studies were conducted across various geographical regions as defined by the World Bank [27], that is, sub-Saharan Africa (n=13), East-Asia and the Pacific (n=8), Europe and Central Asia (n=7), Middle-East and North Africa (n=4), Latin America and Caribbean (n=2), North America (n=5), and inter-region (n=6) (Table 1).

**Table 1:** Characteristics of included studies * indicates based on World Bank classification. ** indicates addressing lymphedema in general, irrespective of causative agents.

Study region*, author, year	Country	Intervention/investigation	Focus	Study design	Lymphedema cause	Affected limb
Sub-Saharan Africa						
Churko et al., 2021 [21]	Ethiopia	Knowledge assessment	Knowledge	Cross-sectional	Podoconiosis	Lower limb
Debrah et al., 2024 [28]	Ghana	Stringent hygiene measures using the essential package of care with or without additional administration of doxycycline	Treatment	Randomized, controlled trial	Lymphatic filariasis	Lower limb
Coulibaly et al., 2024 [29]	Mali	Six-week course of doxycycline and intensive hygiene-based care	Treatment	Randomized, controlled trial	Lymphatic filariasis	Lower limb
Ngenya et al., 2024 [30]	Tanzania	Strict hygiene protocols with or without doxycycline	Treatment	Randomized, controlled trial	Lymphatic filariasis	Lower limb
Dellar et al., 2021 [31]	Ethiopia	Short knowledge training for HCWs	Knowledge	Cohort	Lymphatic filariasis	Lower and upper
John et al., 2022 [32]	Tanzania	Evaluation of management practices	Management	Cross-sectional	Lymphatic filariasis	Lower limb
Esubalew et al., 2022 [33]	Ethiopia	Assessment of self-care practices	Management	Cross-sectional	Podoconiosis	Lower limb
Atinbire et al., 2021 [34]	Ghana	training program for integrated lymphedema management	Management	cohort	Lymphatic filariasis	Lower limb
Douglass et al., 2020 [35]	Ethiopia	Lymphatic stimulating self-care practices	Management	Cluster randomized controlled trial	Lymphatic filariasis	Lower limb
Liakos et al., 2019 [36]	South Africa	Vascularised free lymph node transfer	Treatment	Case report	Cancer-related	Upper limb
Negussie et al., 2018 [37]	Ethiopia	Podoconiosis care and management package	Management	Randomized controlled trial	Podoconiosis	Lower limb
Engdawork et al., 2018 [38]	Ethiopia	Podoconiosis knowledge assessment	Knowledge	Cross-sectional	Podoconiosis	Lower limb
Gabriele et al., 2024 [39]	Italy	Combination of lymphaticovenular anastomosis and liposuction	Treatment	Retrospective observational study	Cancer-related	Lower and upper
East Asia and the Pacific						
Hall et al., 2024 [40]	Sri Lanka	4-week lead-in period of EPC, followed by a 2-week intervention period of daily SSCG (short stretch compression garments) use	Management	Interventional cross-sectional	Lymphatic filariasis	Lower limb
Saha et al., 2023 [41]	India	Assessment of self-care knowledge	Knowledge	Cross-sectional observational study	Lymphatic filariasis	Lower limb
Narahari et al., 2023 [42]	India	Self-care treatment for lymphoedema of lymphatic filariasis using integrative medicine	Treatment	Retrospective data review	Lymphatic filariasis	Lower limb
Mathiarasan et al., 2021 [43]	India	Morbidity management and disability prevention	Management	Retrospective cohort	Lymphatic filariasis	Lower limb
Shetye et al., 2021 [44]	India	Model for self-management	Management	Case report	Lymphatic filariasis	Lower limb
Mues et al., 2018 [45]	India	Anti-fungal cream	Management	Cohort	Lymphatic filariasis	Lower limb
Paramanandam et al., 2022 [46]	India	Compression sleeves	Prevention	Randomized controlled trial	Cancer-related	Upper limb
Douglass et al., 2019 [47]	Bangladesh	Enhanced self-care protocol	Management	Cluster randomized controlled trial	Lymphatic filariasis	Lower limb
Europe and Central Asia						
Uyulmaz et al., 2024 [48]	Switzerland	Immediate lymphovenous anastomosis (LVA) after sarcoma resection	Prevention	Quasi-experimental and literature review	Cancer-related	Lower limb
Bordianu et al., 2024 [49]	Romania	Vascularized omentum lymph node transfer (VOLNT)	Treatment	Retrospective observational study	Cancer-related	Upper limb
Do et al., 2017 [50]	Korea	Bandaging	Management	Case study	Cancer-related	Upper limb
Taradaj et al., 2014 [51]	Poland	Kinesio taping	Treatment	Case report	Cancer-related	Upper limb
Malicka et al., 2014 [52]	Poland	Kinesio taping	Treatment	Case-control	Cancer-related	Upper limb
Gallagher et al., 2022 [53]	Ireland	Adjustable compression sleeve	Management	Case study	Cancer-related	Upper limb
Ochalek et al., 2017 [54]	Austria	Compression sleeves	Prevention	Randomized controlled trial	Cancer-related	Upper limb
Middle East and North Africa						
Özçete and Eyigör, 2020 [55]	Türkiye	Kinesio taping and low-level laser therapy	Treatment	Case report	Cancer-related	Upper limb
Feldman et al., 2015 [56]	Turkiye	Lymphatic Microsurgical Preventive Healing Approach (LYMPHA)	Prevention	Cohort	Cancer-related	Upper limb
Askary and Elshazly, 2022 [57]	Egypt	Cryotherapy	Treatment	RCT	Cancer-related	Upper limb
Tantawy et al., 2019 [58]	Egypt	Kinesio taping and pressure garment comparison	Treatment	Randomized controlled trial	Cancer-related	Lower and upper
Latin America and Caribbean						
Centeno-Rodriguez and Koshima, 2018 [59]	Costa Rica	Lymphaticovencular anastomosis	Treatment	Case report	Cancer-related	Lower limb
Eddy et al., 2014 [60]	Haiti	Mass Drug Administration	Management	Cohort	Lymphatic filariasis	Lower limb
North America						
Wright et al., 2023 [61]	USA	comparing PCD (pneumatic compression devices) plus conservative care (PCD+CC) to CC alone	Management	Randomized, controlled trial	Non-specific**	Lower limb
Leard and Barrett, 2014 [62]	USA	Complete Decongestive Therapy	Treatment	Case report	Obesity	Lower limb
Cebicci et al., 2016 [63]	USA	Extracorporeal Shock Wave Therapy	Treatment	Prospective clinical pilot study	Cancer-related	Upper limb
Tan and Wilson, 2019 [64]	USA	Physical therapy	Management	Case report	Cancer-related	Upper limb
Chun et al., 2022 [65]	USA	Immediate lymphatic reconstruction	Prevention	Systematic review and meta-analysis	Cancer-related	Lower and upper
Inter-region						
Baumann et al., 2018 [66]	-	Exercise	Prevention	Systematic review	Cancer-related	Upper limb
Baxter, 2017 [67]	-	Low-level laser therapy (photobiomodulation therapy)	Treatment	Systematic review	Cancer-related	Upper limb
Douglass et al., 2016 [68]	-	Self-care	Management	Systematic review	Lymphatic filariasis and cancer-related	Lower and upper
Stocks et al., 2015 [69]	-	Hygiene-based lymphedema management	Management	Systematic review	Lymphatic filariasis	Lower limb
Hayes et al., 2021 [70]	-	Exercise	Prevention and Treatment	Systematic review	Cancer-related	Lower and upper
Kong et al., 2022 [71]	-	Microsurgery techniques	Treatment	Systematic review	Cancer-related	Lower and upper limbs

With respect to the anatomical focus of the studies, 23 studies focused on interventions for lower limb lymphedema, 15 focused on upper limb lymphedema, and seven focused on both lower and upper limb lymphedema. In terms of the intervention focus of the studies, 18 focused on treatment interventions, seven on preventive interventions, 17 on management interventions, and four on knowledge interventions.

Preventive strategies for secondary lower or upper limb lymphedema and their effectiveness

Seven studies in this review explored preventive strategies for secondary lymphedema that affects either the lower or upper limbs, particularly lymphedema secondary to cancer treatment or surgery. These strategies included surgical interventions such as lymphatic reconstruction and physical therapies such as exercise and compression sleeves.

Exercise

Baumann et al. (2018) conducted a systematic review to examine whether exercise has a preventive effect on secondary lymphedema in breast cancer patients following treatment [66]. The findings indicated that women who participated in structured postoperative exercise programs had a lower incidence of secondary lymphedema than did those who remained inactive. Specifically, the review highlighted that resistance training and aerobic exercise did not increase the risk of lymphedema; instead, they played crucial roles in maintaining lymphatic flow. While the outcomes varied across the studies included in this review, onset as well as diagnosis of lymphedema were significantly decreased in five studies in the exercise group, and the overarching conclusion of the review was that exercise serves as a safe and effective preventive measure for secondary lymphedema in breast cancer survivors [67].

In another systematic review of 12 studies encompassing 1,955 participants, Hayes et al. (2022) demonstrated that exercise significantly reduced the relative risk of developing secondary lymphedema [70]. The overall relative risk for participants in the exercise group compared with the non-exercise group was 0.90 (95% confidence interval (CI): 0.72-1.13), indicating a 10% reduction in lymphedema risk. Importantly, for individuals who had five or more lymph nodes removed, the preventive effects of exercise were even more pronounced, with a relative risk of 0.49 (95% CI: 0.28-0.85). These findings suggest that for high-risk individuals, structured physical activity could reduce their risk of developing lymphedema by more than 50% [70].

Compression Sleeves

Studies exploring compression sleeves as a preventive strategy for secondary lymphedema suggest that they are effective in reducing arm swelling and the risk of developing lymphedema. Paramanandam et al. (2022) conducted an RCT to investigate whether the prophylactic use of compression sleeves could prevent secondary lymphedema in women who had undergone axillary lymph node dissection (ALND) for breast cancer [46]. The hazard ratio (HR) for arm swelling in the compression group relative to the control group was 0.61 (95% CI: 0.43-0.85, p = 0.004) according to bioimpedance spectroscopy (BIS) measurements, indicating a 39% reduction in risk. Using relative arm volume increase (RAVI) measurements, the HR was 0.56 (95% CI: 0.33-0.96, p = 0.034), suggesting a 44% reduction in risk. At one-year post-surgery, the cumulative incidence of arm swelling was 42% in the compression group compared to 52% in the control group based on BIS, and 14% in the compression group compared to 25% in the control group according to the RAVI [46]. These results demonstrate that compression sleeves not only reduce the incidence of lymphedema but also delay its onset in high-risk breast cancer patients.

Ochalek et al. (2017) conducted another RCT to evaluate whether early postoperative compression therapy could prevent the development of secondary lymphedema [72]. The results revealed that postoperative arm swelling was significantly lower in the compression group compared to the control group. At one-year post-surgery, the average change in excess volume was -67.6 mL in the compression group versus +114.5 mL in the control group (p < 0.001). This finding indicates that women who used compression sleeves had significantly less arm swelling than did those who did not.

Lymphatic Reconstruction

Lymphatic reconstruction techniques, including Lymphatic Microsurgical Preventive Healing Approach (LYMPHA), Immediate Lymphatic Reconstruction (ILR), and Primary Lymphovenous Anastomosis (PLVA), have demonstrated significant effectiveness in preserving lymphatic function and reducing postoperative lymphedema rates. Feldman et al. (2015) conducted a study on the feasibility, safety, and effectiveness of the LYMPHA in preventing breast cancer-related lymphedema (CRL) in patients undergoing ALND [56]. The results revealed that only 12.5% (3 out of 24) of the LYMPHA patients developed lymphedema, whereas 50% (four out of eight) in the control group developed lymphedema. These findings suggest that LYMPHA significantly reduces the risk of secondary lymphedema. Additionally, no LYMPHA-related complications were reported, indicating that LYMPHA is a promising preventive intervention [56]. In a meta-analysis to assess the effectiveness of ILR in preventing secondary lymphedema among patients undergoing surgical procedures such as ALND, Chun et al. (2022) demonstrated that ILR, particularly the LYMPHA technique, significantly reduced the incidence of secondary lymphedema [65]. The findings indicated that of 789 patients, only 2.7% developed lymphedema in the upper extremity, whereas 3.6% developed lymphedema in the lower extremity [66]. Similarly, Uyulmaz et al. (2024) assessed the effectiveness of immediate lymphovenous anastomosis (LVA) following sarcoma resection to prevent secondary lymphedema in lower limbs. This quasi-experimental study revealed that patients who underwent LVA had a 60% lower risk of developing lymphedema than those who did not, with only 10% of LVA patients developing lymphedema compared to 25% in the control group over a one-year follow-up [48]. The study reported a 36% postoperative lymphedema rate [48].

However, the authors of these three studies noted that further studies are required to confirm the sustained effectiveness of these surgical techniques for the prevention of secondary lymphedema [48,56,65].

Treatment strategies for secondary lower or upper limb lymphedema and their effectiveness

This review included 18 studies exploring treatment strategies for secondary lymphedema that affect either the lower or upper limbs. The identified treatment strategies are categorized under four main approaches: surgical interventions, such as lymphaticovenular anastomosis (LVA), common for CRL; hygiene-based treatments, often used for filarial lymphedema; adjunct therapies, like laser therapy and Kinesio taping (KT) [73], and compression or physical treatments.

Hygiene-Based and Self-Care Treatment

Three studies explored the combination of hygiene-based interventions and pharmaceutical treatments for patients with filarial lymphedema. These RCTs investigated the effects of strict hygiene protocols with or without doxycycline on treatment outcomes for filarial lymphedema [74]. All participants were initiated into a program of cleaning the affected limbs. Debrah et al. (2024) in Ghana indicated leg lymphedema improvement was more common than worsening, i.e., 20% improvement in a group that was given 200 mg doxycycline in additional to the hygiene intervention, 19.5% in a group that was given 100 mg doxycycline and 27.4% in the placebo group [28]. Coulibaly et al. (2024) in Mali reported a 41% improvement in the doxycycline arm, 37% improvement in the placebo arm [29]. In Tanzania, Ngenya et al. (2024) showed that after 24 months, 17.7% of all participants displayed improved limb conditions, including 14.4% in the doxycycline 200 mg group, 15.2% in the doxycycline 100 mg group, and 23.4% in the placebo group [30].

Complete Decongestive Therapy (CDT)

In a case study of obesity-related lymphedema, Leard and Barrett (2014) reported that CDT in an outpatient setting resulted in a 60% decrease in the patient's lower limb [62].

Physical Treatments

In a meta-analysis of 36 studies with 1,741 participants to evaluate the effects of exercise on the treatment of CRL, Hayes et al. (2021) noted that exercise interventions led to improvements in pain, upper-body function and strength, lower-body strength, fatigue, and quality of life, with standardized mean differences ranging from 0.3 to 0.8 (p < 0.05) [70].

Surgical and Microsurgical Interventions

Surgical interventions are commonly used for CRL, aiming to restore lymphatic function through lymph node transfers and LVA [75,76].

LVA and Liposuction for Treatment

Gabriele et al. (2024) observed that after LVA and liposuction for treatment of secondary lymphedema, one-year post-surgery results revealed an average volume reduction of 37.9%, a decrease in the lymphangitis rate from 4.67 to 0.95 per year and improvement in the quality-of-life score from 68.7 to 16 according to the LLIS scale [39]. The study concluded that the combination of LVA and liposuction represents a valid, minimally invasive, and well-tolerated strategy for treating CRL, ensuring stable results over time. Additionally, Centeno-Rodriguez and Koshima (2018) presented a case study in which a patient with lower limb CRL underwent LVA [59]. Ten months post-surgery, the patient exhibited a 12.5 cm reduction in limb circumference 10 cm above the knee, a 9 cm reduction at the knee, a 3 cm reduction 10 cm below the knee, and a 0.5 cm reduction at the dorsum of the foot [60].

Vascularized Free Lymph Node Transfer (VLNT) and Lymphovenous Bypass (LVB)

Liakos et al. (2019) in South Africa presented a case report on VLNT for upper limb CRL. Compared with preoperative measurements, follow-up circumferential limb measurements of the hand, 5 cm above the wrist, 15 cm above the wrist, and 10 cm above the elbow at six months after surgery showed slight improvements, averaging approximately 1.1 cm in the affected limb [36].

In a meta-analysis of 37 studies, Kong et al. (2022) assessed the effectiveness of LVB and VLNT in treating lymphedema, demonstrating that microsurgical techniques significantly outperform conservative treatments. Patients who underwent microsurgery were seven times more likely to achieve excellent results (odds ratio (OR) = 7.07), with an average limb circumference reduction of 44.68% and a volume reduction of 38.32%. While complications such as lymphatic leakage (32%) were noted for both VLNT and LVB, VLNT had a lower failure rate (8%) than did LVB (12%) [71].

Vascularized Omentum Lymph Node Transfer (VOLNT)

In a retrospective study, Bordianu et al. (2024) found that after performing VOLNT, the volume difference between the upper limbs decreased from 15% to 5%, hence, an overall decrease of 66% [49]. Most of the patients whose upper limbs were measured at one-year follow-up registered a 1 cc circumference reduction (47%) [49].

These studies collectively demonstrate that microsurgical techniques, including LVA, VLNT, and VOLNT, significantly reduce limb volume and improve patient-reported outcomes, particularly in CRL cases.

Non-surgical and Non-hygiene-Based Therapies

These non-surgical and non-hygiene-based therapies, such as cryotherapy, KT, low-level laser therapy, and integrative medicine (IM) approaches like yoga and manual lymphatic drainage, have been identified to serve as complementary treatments alongside conventional therapies.

IM approaches: Narahari et al. (2023) in India conducted a retrospective data review exploring IM approaches for lymphatic filariasis-related lymphedema [42]. The IM treatment for lymphoedema used a combination of Indian traditional medicine, Ayurveda, alongside yoga exercises, compression therapy, antibiotics, and antifungal treatments. The study indicated that limb volume was reduced by 24.5% following an intensive supervised care period of approximately 14 days. Limb volume further reduced by 1.42% after 81.45 days, and by 2.3% between the first and second follow-up visits at 231 days. Only 4% of patients exhibited new bacterial entry points at the first follow-up. Health-related quality of life increased by 17.8 at the first follow-up and 18.6 at the second follow-up. No patients developed new cellulitis episodes at the first follow-up, and only 5.3% developed new episodes of cellulitis at the second follow-up.

KT: Tantawy et al. (2019) compared KT and pressure garments in an RCT involving both upper and lower limb lymphedema patients [58]. The sum of limb circumferences, Shoulder Pain and Disability Index (SPADI), hand grip strength, and quality of life significantly improved after treatment with KT (p < 0.05) as compared to pressure garments which showed no significant improvement in SPADI, hand grip strength, physical, role, pain, and fatigue score (p > 0.05). Özçete and Eyigör (2020) examined KT combined with low-level laser therapy for upper limb CRL [55]. This case report found that the combination therapy resulted in an arm volume decrease from 691 mL to 454 mL after the treatments [56]. Malicka et al. (2014) conducted a pilot study to evaluate the effects of KT on early-stage lymphedema (stage I) in women who had undergone axillary lymphadenectomy for breast cancer [52]. After the intervention period, the KT group demonstrated a statistically significant reduction in lymphedema extent (p = 0.0009), whereas the control group showed no significant improvement (p = 0.36). These findings indicate that KT may be beneficial in the early stages of lymphedema, providing a non-invasive and well-tolerated intervention for patients who may not be candidates for compression-based therapies [52].

Similarly, Taradaj et al. (2014) presented a case study of a patient with breast CRL whose treatment consisted of manual lymphatic drainage, pneumatic compressions, and KT [51]. They noted that KT had a significant accelerating effect on the reduction of lymphedema. During the first four days of therapy with manual lymphatic drainage and intermittent pneumatic compression, lymphedema was reduced by 31 cm^3^; however, after a three-day application of K-tapes, the observed reduction was by 194 cm^3^. Similarly, after standard treatment on day 18, a reduction was only 40 cm^3^, and after application of KT, we observed again acceleration in oedema reduction by 105 cm^3^ [51].

Low-level laser therapy (photobiomodulation therapy): Additionally, in a systemic review on the effectiveness of low-level laser therapy (photobiomodulation therapy) in CRL, Baxter et al. (2017) reported strong evidence (three high quality trials) showing lower-laser therapy as an effective for lymphedema evidenced by limb circumference/volume reduction at a short-term follow-up. The study also indicated that low-laser therapy was effective for short-term pain relief [67].

Cryotherapy: Askary and Elshazly (2022) investigated cryotherapy for post-mastectomy upper limb lymphedema in an RCT [57] in which traditional physical therapy programs (manual lymphatic drainage, pneumatic compression, bandaging breathing exercises, circulatory exercises, shoulder mobilizations, and range of motion (ROM) exercises) were combined with pulsed local cryotherapy three times per week. For 12 weeks, Group (B) received only traditional physical therapy three times per week. Results revealed a significant effect of treatment and time on the thickness and circumferential measurement of the wrist, below the elbow, and above the elbow (p = 0.001), indicating that cryotherapy is an effective adjunct modality for the treatment of secondary lymphedema [57].

Extracorporeal shock wave therapy (ESWT): Cebicci et al. (2016) explored ESWT as a treatment for upper limb lymphedema, particularly in post-mastectomy patients [63]. The findings revealed a significant reduction of lymphedema with ESWT treatment in all patients, maintained over a six-month follow-up period. The mean volume displacement of the affected upper extremity before treatment was 870.45 ± 384.19 mL at six months, and after the treatment, it was 604.54 ± 381.74 mL. Additionally, a marked improvement was observed in the functional status and quality of life of study patients [63].

Management strategies for secondary lower or upper limb lymphedema and their effectiveness

This review included 17 studies exploring management strategies for secondary lymphedema. The studies in this review highlight self-care and hygiene-based management for filarial and podoconiosis-related lymphedema, compression garments, MDA and MMDP, adjunct management strategies, such as antifungal treatments, and for filarial lymphedema.

Self-Care and Hygiene-Based Management Strategies

In a systematic review of 22 studies, Stocks et al. (2015) noted that participation in hygiene-based lymphedema management was associated with a lower incidence of acute dermatolymphagioadenitis (ADLA) (OR 0.32) [69].

Douglass et al. (2020) conducted a cluster-RCT in Ethiopia evaluating lymphatic-stimulating self-care practices in patients with moderate-to-severe filarial lymphedema [36]. Over 24 weeks, patients performing diaphragmatic breathing, self-massage, and muscle exercises saw a 42.4% reduction in severe lymphedema cases, with only 12% reporting acute attacks (p = 0.014) compared to the 37.8% reduction of lymphedema and 38% reporting acute attacks in the control groups [35].

In a systematic review to evaluate the evidence for effective lymphedema self-care strategies for secondary lymphedema, Douglass et al. (2016) indicated that hygiene-centered self-care reduced the frequency and duration of acute episodes by 54% in filariasis-related lymphedema, and in CRL, home-based exercise, including deep breathing, delivered significant volume reductions over standard self-care alone [68].

Shetye et al. (2021) presented a case study where a patient with 18 years of filarial lymphedema underwent a 20-day self-management program incorporating aerobic exercises, decongestive therapy, and limb elevation [44]. The percentage difference in excess limb volume reduced from 88.54% to 7.37% after the 20-day self-management program. The patient reported improved function over eight months, highlighting structured self-care as an effective approach [45]. Similarly, Douglass et al. (2020) evaluated an enhanced self-care protocol that included lymphatic massage, deep breathing, and dietary recommendations [47]. Participants in the intervention group, who practiced lymphatic massage, deep breathing, and thigh exercises, showed a greater reduction in severe lymphedema cases (-42.4%) compared to the control group (-37.8%). The intervention also led to significantly fewer acute attacks - only 12% of participants in the intervention group experienced acute attacks compared to 38% in the control group (p = 0.014) [47].

Negussie et al. (2018) assessed the implementation of an intervention focused on promoting self-care practices, including regular foot hygiene, use of antiseptics, application of emollients, and wearing shoes and socks to prevent soil exposure [37]. The study reported significant improvements in lymphedema severity and a reduction in the frequency of acute inflammatory episodes among participants adhering to the self-care regimen. The incidence of acute dermatolymphangioadenitis (ADLA) was 19·4 episodes per person-year (95% CI 18.9-19.9) in the intervention group compared to 23.9 episodes per person-year (23.4-24.4) in the control group [34].

Atinbire et al. (2021) highlighted that hygiene training for lymphedema patients significantly improved self-care behaviors, with 51% of participants washing their affected limbs more than once a day [34]. This practice reduced the frequency of acute episodes by 20%, from 54% to 34% after the training [34].

Despite the proven importance of hygiene and self-care practices in management of filarial lymphedema, a study conducted in Tanzania, by John et al. (2022) found that 78.4% of patients practiced inappropriate hygiene care such as washing the affected limbs once a day (46%), lack of entry lesion inspection (83.8%), and inappropriate use of footwear (86.5%). Similarly, in Ethiopia, Esubalew et al. (2022) noted that only 10.8% of the patients washed their feet with soap, and about 70.8% wore shoes, and overall, only 64% practiced self-care [33].

Compression Garments and Sleeves

Compression garments have been shown to effectively reduce limb volume. Hall et al. (2024) in Sri Lanka assessed the effectiveness of short-stretch compression garments (SSCG) for filarial lymphedema [40]. The study revealed that the use of SSCG resulted in a median limb volume reduction of 11.3%, supporting their role in managing lymphedema in low-resource settings [40].

Wright et al. (2023) demonstrated that using pneumatic compression devices (PCD) in combination with conservative care (CC) led to a 24% reduction in leg circumference compared to a -2.5% change with CC alone, proving their efficacy in lymphedema management [61]. Additionally, the use of adjustable compression sleeves in a case study showed improvements in skin condition and volume reduction during the intensive phase of treatment [54].

In the study by Do et al. (2017), the researchers examined the effects of bandaging with an additional pad and taping on post-mastectomy upper limb lymphedema [50]. The intervention led to a 2.4 cm reduction in arm circumference and a 10.2% decrease in fluid retention, along with improved limb function and reduced discomfort [52]. These findings suggest that combining bandaging, padding, and taping may enhance lymphatic drainage, though further research is needed to validate its effectiveness on a larger scale.

Adjunct Management Strategies

Mues et al. (2018) investigated the impact of antifungal cream use on episodes of ADLA among lymphedema patients [45]. Their findings revealed that for each additional month a patient adhered to daily antifungal cream application over 12 months, there was a 23% reduction in the number of ADLA episodes at the 18-month follow-up (rate ratio = 0.77; 95% CI: 0.62 to 0.96) [45].

Physiotherapy and mechanical stimulation therapies were identified to be used in the treatment of both filarial and CRL. Tan and Wilson (2019) investigated the role of structured physical therapy programs in managing upper limb CRL [64]. The patient demonstrated a greater than 1% reduction in swelling [64].

MDA and MMDP

The role of MDA and MMDP in reducing lymphedema-related morbidity has been highlighted in studies by Eddy et al. (2014) and Mathiarasan et al. (2021). Eddy et al. (2014) showed that MDA did not change the progression of lymphedema in terms of stage transitions, nor did it decrease ADL (acute dermato-lymphangio-adenitis) episodes [60]. The study revealed, though, that patients who received MDA reported improvement in four areas of lymphedema-related quality of life (p ≤ 0.01).

Mathiarasan et al. (2021) revealed that MMDP did not provide significant improvement in filarial lymphedema [43]. The overall reversal and progression were observed in 13% and 52% of cases, respectively. This study has revealed that the second arm of the Global Programme to Eliminate Lymphatic Filariasis (GPELF), MMDP, did not yield the desired results as evidenced by the advancement of lymphedema grades [43].

Causes of lower and upper limb secondary lymphedema globally

Based on the studies included in this review, the causative agents of lower and upper limb secondary lymphedema can be categorized into three primary etiologies: lymphatic filariasis [28-30], podoconiosis, and CRL. Lymphatic filariasis was reported as the leading cause of lower limb lymphedema in sub-Saharan Africa (Ghana, Mali, Tanzania, and Ethiopia) as shown by studies from Debrah et al. (2024) [28], Coulibaly et al. (2024) [29], Ngenya et al. (2024) [30], John et al. (2022) [32], Dellar et al. (2021) [31], and Douglass et al. (2020) [35]. Lymphatic filariasis is also reported as a cause of secondary lymphedema in East Asia and the Pacific (Sri Lanka, India, and Bangladesh) by Hall et al. (2024) [40], Saha et al. (2023) [41], Narahari et al. (2023) [42], and Douglass et al. (2019) [74]. Additionally, Leard and Barrett (2014) in the USA presented a case of a patient with chronic stage III lymphedema secondary to morbid obesity [62].

Podoconiosis was identified in Ethiopia as a prevalent cause of lower limb lymphedema, attributed to chronic exposure to silica-rich soils. Esubalew et al. (2022) [33], Churko et al. (2021) [21], and Negussie et al. (2018) [37] all documented podoconiosis as a major contributor to lymphedema cases in rural highland regions. These studies emphasize the non-infectious yet preventable nature of podoconiosis, which primarily affects individuals without protective footwear.

CRL emerged as the predominant cause of both upper and lower limb lymphedema in Europe, Central Asia, the Middle East, and North America. Studies in Switzerland (Uyulmaz et al., 2024 [48]), Romania (Bordianu et al., 2024 [49]), and Poland (Taradaj et al., 2014 [51]; Malicka et al., 2014 [52]) reported upper limb lymphedema following breast cancer treatment, particularly after ALND and radiation therapy. Similar findings were reported in Türkiye (Feldman et al., 2015 [56]), Egypt (Askary and Elshazly, 2022 [57]), and the USA (Chun et al., 2022 [65]), while cancer-related lower limb lymphedema was observed in Italy (Gabriele et al., 2024 [39]) and Costa Rica (Centeno-Rodriguez and Koshima, 2018 [59]).

These studies highlight regional variations in secondary lymphedema causes, with lymphatic filariasis dominating in endemic tropical areas [51], podoconiosis being geographically confined to rural highland regions with silica-rich soils, and CRL being a major concern in high-income regions with advanced oncology treatments.

Knowledge of secondary lymphedema

This review included four studies exploring knowledge of secondary lymphedema and its prevention, treatment, and management.

Patient Knowledge

Saha et al. (2023) noted limited knowledge of self-care practices for filarial lymphedema in India. They noted that only about 26.1% correctly knew the management of the affected area [41].

Health Providers’ Knowledge

A study conducted in northwest Ethiopia by Dellar et al. (2022) assessed the knowledge, attitudes, and practices of healthcare workers regarding lymphedema management. They noted that before interventional training, 71% of healthcare workers held at least one stigmatizing attitude toward lymphedema patients, which only slightly improved to 66% after training. Furthermore, many healthcare professionals were unable to correctly identify ways to prevent or treat the disease, highlighting the urgent need for targeted training interventions [31].

Churko et al. (2021) noted that 23.1% of health professionals had poor knowledge of podoconiosis, and the majority lacked clinical experience with treating patients. Findings revealed that 21.9% of healthcare professionals incorrectly identified podoconiosis as an infectious disease, and only 11.6% had treated podoconiosis patients [21].

Community Knowledge

Engdawork et al. (2018) noted that misconceptions about podoconiosis were widespread among youth, with half of the participants believing it was contagious. Misunderstandings about its causes, including the belief in the "evil eye" or snake bites, were also prevalent [38]. However, a significant proportion of youth (82%) correctly identified barefoot walking as a trigger for the disease. This study indicates that while youth have the potential to become effective advocates for accurate information about podoconiosis, efforts are needed to challenge their inaccurate beliefs about contagion and help link genetic and environmental factors to preventive actions [38].

Discussion

This scoping review identified and synthesized global preventive and management strategies for secondary lymphedema, highlighting their effectiveness and application across different contexts. The findings emphasize the importance of early intervention in prevention, as well as comprehensive and multi-modal approaches in management to improve patient outcomes. Additionally, the role of knowledge and awareness in influencing patient adherence and healthcare provider practices was evident, underscoring the need for structured education and training programs.

Surgical interventions such as LVA and VLNT have been implemented and assessed for the prevention and treatment of secondary lymphedema. This review found a significant reduction in limb volume following these procedures, aligning with recent findings by Yamamoto (2024), who reported that super microsurgical LVA achieved notable improvements in early-to-moderate stage lymphedema [75]. The LYMPHA has also been highlighted in recent research as a preventive measure for breast CRL, demonstrating a reduced incidence in patients undergoing ALND. However, accessibility remains a key barrier, with McNeely et al. (2024) emphasizing the disparities in microsurgical intervention availability, particularly in low-resource settings [77].

Compression therapy has been extensively studied for its role in reducing swelling and preventing lymphedema progression. Studies included in this review, such as Paramanandam et al. (2022) and Ochalek et al. (2017), reported that compression sleeves reduced swelling incidence. Similar efficacy was reported by Meyer et al. (2024), who found that compression therapy, combined with physical therapy, significantly reduced limb swelling in post-cancer patients [78]. However, adherence continues to be a challenge. A cross-sectional survey by Klugman et al. (2024) found that a substantial proportion of breast cancer survivors lacked adequate knowledge about lymphedema prevention and management, contributing to low compliance with compression therapy recommendations [79].

Exercise-based interventions have increasingly been integrated into lymphedema management. This review found that structured physical activity reduced the risk of secondary lymphedema in high-risk individuals. Grgić et al. (2024) corroborated this, demonstrating that resistance and aerobic exercise programs significantly improved lymphatic function and quality of life in breast cancer survivors.

Hygiene-based interventions, particularly in regions affected by lymphatic filariasis and podoconiosis, remain crucial for lymphedema prevention. Bisrat et al. (2024) similarly reported a decline in acute dermato-lymphangio-adenitis incidence following the implementation of large-scale hygiene and self-care protocols in Ethiopia [80]. However, disparities in access to clean water and hygiene supplies persist, as highlighted by Chiphwanya et al. (2024), who noted that many endemic communities still struggle with the infrastructure needed to sustain such preventive measures [81]. Constantinovici et al. (2024) found similar success with CDT in reducing swelling and improving functional outcomes in post-traumatic lymphedema patients [82]. However, its high cost and limited availability of trained therapists remain significant challenges.

Emerging adjunct therapies such as KT and low-level laser therapy have also demonstrated promise. Yang et al. (2024) and Tremback-Ball et al. (2018) supported these findings, showing that KT enhanced lymphatic flow and reduced discomfort in post-mastectomy patients [83]. However, these interventions should not replace conventional therapies but rather be integrated into comprehensive treatment plans, as recommended by Wong et al. (2024) [76].

This study examines the feasibility of implementing existing preventive and management strategies for secondary lymphedema within low- and middle-income settings. The review highlights a range of interventions, from low-cost, community-based approaches to more resource-intensive surgical and therapeutic techniques [84,85]. The applicability of these strategies in low- and middle-income country (LMIC) settings depends on factors such as cost, infrastructure, and the availability of trained healthcare personnel.

Preventive strategies such as lymphatic reconstruction through microsurgical techniques have demonstrated effectiveness in reducing post-surgical lymphedema [49,57,66], yet their feasibility remains low in LMICs due to limited surgical expertise and high procedural costs.

Management strategies such as self-care and hygiene-based interventions have demonstrated high feasibility and effectiveness in LMICs [86,87], particularly in controlling filarial and podoconiosis-related lymphedema. Douglass et al. (2020) reported that lymphatic-stimulating self-care practices reduced acute inflammatory episodes [68], while Negussie et al. (2018) [37] and Esubalew et al. (2022) [33] found that consistent foot hygiene and protective footwear significantly reduced inflammation in podoconiosis patients [88,89]. However, John et al. (2022) found that only 21.8% of affected individuals practiced consistent self-care [32], indicating the need for targeted education programs to improve adherence.

Additionally, compression therapy [41,62], which has been shown to reduce limb volume by 11.3-24%, is feasible in low-income settings where a trained therapist for filarial lymphedema may not be available [41]. These findings highlight that low-cost, community-led interventions like self-care and hygiene-based management are more feasible than equipment-intensive therapies in LMICs.

While MDA and MMDP have been effectively implemented in LMICs, Eddy et al. (2014) [60] and Mathiarasan et al. (2021) [43] have shown that they have an insignificant effect on the reduction of acute inflammatory episodes and improvement of lymphedema.

Despite the proven effectiveness of many strategies, their implementation in LMICs is hindered by financial constraints, limited healthcare infrastructure, and low adherence rates. While adjunct treatments like antifungal therapy [46] and physiotherapy have demonstrated 23% and 30% reductions in lymphedema symptoms, respectively, their feasibility depends on availability and access to trained professionals. Additionally, cultural perceptions regarding lymphedema causation and treatment, that is, misconceptions about genetic or supernatural causes, do persist and impact adherence, particularly in podoconiosis-affected communities. To improve feasibility, it is essential to expand community education programs, subsidize affordable interventions, train healthcare workers, and integrate lymphedema care into existing health services. Ultimately, self-care and hygiene-based strategies remain the most scalable approaches, while high-cost medical interventions will require long-term investment in healthcare infrastructure and human resources to be feasible in LMICs.

## Conclusions

This scoping review highlights the diverse preventive, treatment, and management strategies for secondary lymphedema across various global contexts. While microsurgical interventions such as LVB and VLNT show promise in reducing disease progression, their high cost and limited availability in low-resource settings remain a challenge. In contrast, hygiene-based interventions and self-care practices are the most accessible and scalable approaches, particularly for lymphatic filariasis and podoconiosis-related lymphedema. Compression therapy and adjunct treatments like low-level laser therapy, KT, and cryotherapy provide additional benefits but require better adherence and awareness to maximize effectiveness. Despite advancements, significant gaps remain, particularly in LMICs, where limited access to care, healthcare infrastructure, and financial constraints hinder the implementation of effective interventions. While self-care and hygiene-based strategies offer feasible and cost-effective solutions, high-cost medical interventions will require long-term investment in healthcare infrastructure and workforce development to ensure broader accessibility. Additionally, long-term studies are needed to evaluate the sustainability and adherence of various interventions, as most existing research primarily focuses on short-term outcomes. Addressing disparities in access to care, improving health literacy, and integrating lymphedema management into existing health systems will be crucial for reducing the global burden of secondary lymphedema and improving patient outcomes.
